# Applying negative sample denoising and multi-view feature for lncRNA-disease association prediction

**DOI:** 10.3389/fgene.2023.1332273

**Published:** 2024-01-09

**Authors:** Dengju Yao, Bo Zhang, Xiangkui Li, Xiaojuan Zhan, Xiaorong Zhan, Binbin Zhang

**Affiliations:** ^1^ School of Computer Science and Technology, Harbin University of Science and Technology, Harbin, China; ^2^ College of Computer Science and Technology, Heilongjiang Institute of Technology, Harbin, China; ^3^ Department of Endocrinology and Metabolism, Hospital of South University of Science and Technology, Shenzhen, China

**Keywords:** lncRNA-disease association, negative sample denoising, multi-view feature, stacking ensemble learning, graph attention networks

## Abstract

Increasing evidence indicates that mutations and dysregulation of long non-coding RNA (lncRNA) play a crucial role in the pathogenesis and prognosis of complex human diseases. Computational methods for predicting the association between lncRNAs and diseases have gained increasing attention. However, these methods face two key challenges: obtaining reliable negative samples and incorporating lncRNA-disease association (LDA) information from multiple perspectives. This paper proposes a method called NDMLDA, which combines multi-view feature extraction, unsupervised negative sample denoising, and stacking ensemble classifier. Firstly, an unsupervised method (K-means) is used to design a negative sample denoising module to alleviate the imbalance of samples and the impact of potential noise in the negative samples on model performance. Secondly, graph attention networks are employed to extract multi-view features of both lncRNAs and diseases, thereby enhancing the learning of association information between them. Finally, lncRNA-disease association prediction is implemented through a stacking ensemble classifier. Existing research datasets are integrated to evaluate performance, and 5-fold cross-validation is conducted on this dataset. Experimental results demonstrate that NDMLDA achieves an AUC of 0.9907and an AUPR of 0.9927, with a 5-fold cross-validation variance of less than 0.1%. These results outperform the baseline methods. Additionally, case studies further illustrate the model’s potential in cancer diagnosis and precision medicine implementation.

## 1 Introduction

Non-coding transcripts, particularly lncRNAs that do not encode proteins, constitute the majority of the genome (Maher, 2012). Typically, lncRNAs are transcripts that exceed 200 nucleotides in length. Noteworthy examples of lncRNAs such as H19 (Brannan et al., 1990) and Xist (Brockdorff et al., 1992) were first implicated in epigenetic regulation in the early 1990s. Numerous functional examples have also demonstrated the involvement of lncRNAs in various human physiological processes, including embryonic stem cell pluripotency, cell cycle regulation, and complex diseases (Rinn and Chang, 2012). Therefore, exploring the relationship between lncRNAs and complex human diseases will contribute to a better understanding of disease pathogenesis and the development of lncRNA-based pharmacology.

In the past decade, extensive studies have identified many types of lncRNAs that can serve as promising biomarkers for cancer diagnosis and targeted therapy. For instance, LINC01608 has been identified as a promising prognostic biomarker for hepatocellular carcinoma (Liu et al., 2022), NALT1 promotes the targeting of PEG10 via sponge microRNA-574-5p to advance colorectal cancer progression (Ye et al., 2022), and RNA demethylase ALKBH5 promotes lung cancer progression (Shen et al., 2022). However, traditional biological experiments used to identify the association between lncRNA and diseases, such as PCR (Heid et al., 1996) and microarray analysis (Zhai et al., 2015), have always been limited by high costs and lack of specificity in exploring and understanding lncRNA.

With advances in computer technology and its ability to handle vast amounts of data, computational method has been explored to validate LDA and has yielded promising results. The first LDA prediction model (called LRLSLDA) was proposed by Chen et al. (Chen and Yan, 2013), utilizing the Laplace regularized least square method to predict LDA. This model is built on the hypothesis that similar diseases are associated with similar lncRNAs (Chen and Yan, 2013). Chen et al. (Chen et al., 2015) enhanced LRLSLDA by introducing a fusion method for lncRNA functional similarity. Although these methods did not achieve excellent prediction performance, they sparked further interest in studying the association between lncRNAs and diseases.

To capture comprehensive association information between lncRNAs and diseases, several LDA prediction methods based on similarity network feature fusion have been proposed. For example, Wei et al. proposed the iLncRNAdis-FB model for data fusion through feature blocks (Wei et al., 2021), Chen et al. proposed the iLDMSF model based on KNN for nonlinear multi-similarity fusion (Chen et al., 2021a), and Fan et al. proposed the GCRFLDA framework that integrates the conditional random field layer and the attention mechanism to fuse various similarities between lncRNAs and diseases in a linear manner as auxiliary features of nodes (Fan et al., 2022).

Moreover, Data sets in Bioinformatics usually present a high level of noise (Miranda et al., 2009). The noisy training data set increases the training time and complexity of the model. Consequently, identifying noisy instances and then eliminating or correcting them are useful techniques in data mining research (Nematzadeh et al., 2020). Chen et al. found that the presence of noisy samples can significantly impact the predictive performance of the LDA model (Chen et al., 2021b) Some papers (Yao et al., 2020; Wei et al., 2021; Kang et al., 2022; Lu and Xie, 2023) have used random sampling to create balanced datasets by including an equal number of unknown and positive samples in an attempt to mitigate the impact of unbalanced datasets. However, this approach may introduce potentially noisy data into the negative sample set. Lan et al. proposed an LDA prediction model based on an improved graph convolution network with Top-K negative sampling (Lan et al., 2021). Another method by Peng et al. involved screening reliable negative samples through a graph autoencoder (Peng et al., 2022). He et al. proposed two similarity-based negative sampling methods, one based on the Euclidean distance calculation between unlabeled samples and positive samples, and the other by reducing the number of unlabeled samples based on the functional similarity between lncRNAs (He et al., 2023).

Although existing methods have achieved good performance in predicting LDA, there still needs to be more potential in utilizing the association information between diseases and lncRNAs. Additionally, constructing the negative sample set may introduce latent LDA as noise, leading to reduced predictive accuracy of the model. This paper proposes a predictive model to construct a more accurate LDA model that combines multi-view feature extraction, an unsupervised negative sample denoising module, and a stacking ensemble classifier to uncover the associations between lncRNAs and diseases. The main contributions of this paper are as follows:

1. To mitigate the impact of sample imbalance and potential noise in negative samples on the model’s performance, a negative sample denoising module is designed using an unsupervised method (K-means (Hartigan and Wong, 1979)). By simultaneously clustering positive and negative samples using K-means, this module not only improves the model’s performance but also provides potential solutions for mitigating sample imbalance and achieving negative sample denoising in LDA.
2. To construct a more precise LDA model, we use graph attention networks (Veličković et al., 2017) to obtain multi-view features. These features are then combined with an unsupervised negative sample denoising module and a stacked ensemble classifier. Experimental results consistently demonstrate the outstanding performance of the proposed LDA prediction model. This model has potential applications in cancer diagnosis and can contribute to the advancement of precision medicine.

## 2 Materials and methods

**FIGURE 1:**
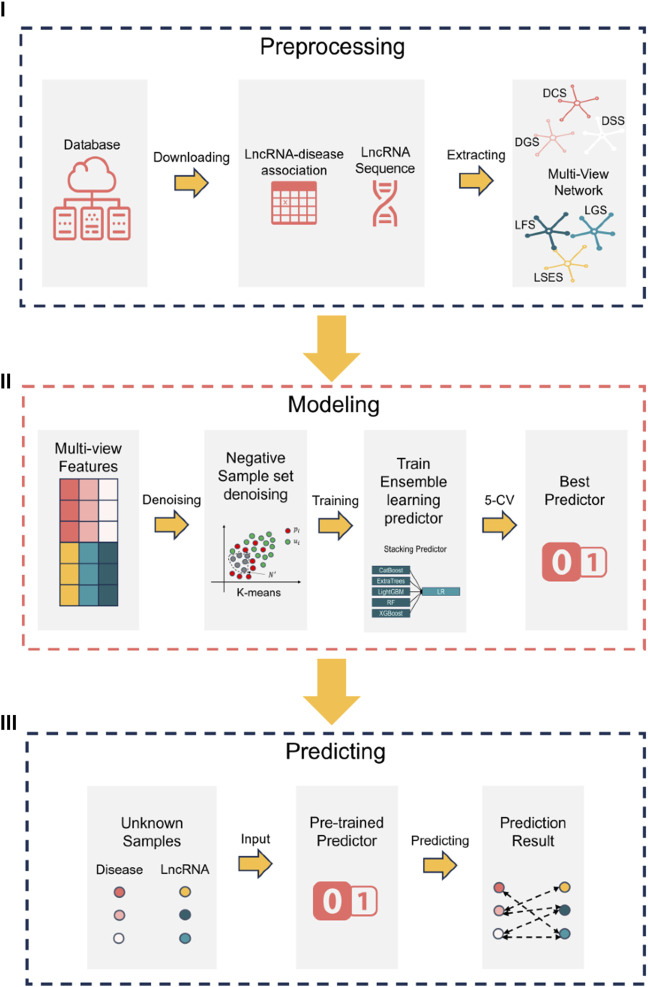
Research flowchart.

The research flowchart of this paper can be divided into three steps, as illustrated in Figure 1 (Ⅰ) data preprocessing (Ⅱ) construction of the NDMLDA model by incorporating multi-view feature extraction, an unsupervised negative sample denoising module, and a stacking ensemble classifier, and (Ⅲ) utilization of the NDMLDA model to make predictions regarding the association between unknown lncRNAs and diseases. Furthermore, in Figure 1 section (Ⅰ), DSS (disease semantic similarity network), DCS (disease cosine similarity network), DGS (disease gaussian interaction profile kernel similarity network), LSES (lncRNA sequence similarity network), LGS (lncRNA gaussian interaction profile kernel similarity network), LFS (LncRNA functional Similarity network) represent six similarity networks, respectively.

### 2.1 Materials

#### 2.1.1 Data source

The data utilized in this research were obtained from five databases: Lnc2Cancer 3.0 (Gao et al., 2021), LncRNADisease v2.0 (Bao et al., 2019), RNADisease v4.0 (Chen et al., 2022), NONCODE v6.0 (Zhao et al., 2021) and lncTarD 2.0 (Zhao et al., 2023). The Lnc2Cancer 3.0 database comprises 9,254 associations between lncRNAs and diseases, involving 2,659 lncRNAs and 216 diseases (Gao et al., 2021). LncRNADisease v2.0 collects 205,959 lncRNA-disease associations, encompassing 19,166 lncRNAs and 529 diseases (Bao et al., 2019). RNADisease v4.0 compiles 11,525 experimentally validated lncRNA-disease associations, encompassing 11,490 lncRNAs and 1,002 diseases (Chen et al., 2022). The NONCODE database includes a total of 96,411 pieces of information regarding non-coding RNA sequences (Zhao et al., 2021). The lncTarD database recruits 8,360 key lncRNA-target regulations associations with 419 disease subtypes, 1,355 lncRNAs, 506 miRNAs, 1,743 protein-coding genes and 286 biological functions.

To gain a more comprehensive understanding of the correlation between lncRNAs and diseases, we merged and manually curated the LDA data from three databases: Lnc2Cancer, LncRNADisease, and RNADisease (see supplementary for details). As a result, we obtained a total of 8,334 lncRNA-disease associations involving 629 lncRNAs and 511 diseases, which were stored in matrix A . Subsequently, we retrieved the sequence information of all lncRNAs in matrix A from the NONCODE database. Additionally, we applied the same preprocessing method to process the data from lncTarD, resulting in 504 lncRNA-disease associations between 103 diseases and 212 lncRNAs. No further data manipulation was performed besides this.

#### 2.1.2 Disease semantics similarity

We use the method proposed by Wang et al. (2010) to calculate the semantic similarity of diseases which is given by the following formula:

$$DSS\left(d_{i},d_{j}\right)=\frac{\sum_{d\in D_{i}\cap D_{j}}\left(SC_{d_{i}}\left(d\right)+SC_{d_{j}}\left(d\right)\right)}{SV_{d_{i}}+SV_{d_{j}}}$$

Where, d represents disease; D represents ancestors’ nodes of d ; ∩ represents intersection; SC and SV represent the semantic contribution value and semantic value of disease, respectively.

#### 2.1.3 Disease cosine similarity

The cosine similarity between two diseases can be calculated using the following formula:

$$DCS\left(d_{i},d_{j}\right)=\frac{A\left(i,:\right)\cdot A\left(j,:\right)}{\left\|A\left(i,:\right)\right\|\times \left\|A\left(j,:\right)\right\|}$$

Where, vector $A\left(i,:\right)$ represents the set of elements in the *i*th row in matrix A . The length of this vector is denoted as $\left\|A\left(i,:\right)\right\|$ .

#### 2.1.4 Disease (lncRNA) Gaussian interaction profile kernel similarity

We utilize the algorithm presented by van Laarhoven et al. (2011) to calculate the similarity of gaussian interaction profile kernel similarity for disease (lncRNA), which is given by the following formulas:

$$DGS=\exp\left(-\gamma_{d}{\left\|A\left(i,:\right)-A\left(j,:\right)\right\|}^{2}\right)$$

$$LGS=\exp\;\left(-\gamma_{l}{\left\|A\left(:,i\right)-A\left(:,j\right)\right\|}^{2}\right)$$

Where, DGS and LGS represents disease (lncRNA) gaussian interaction profile kernel similarity; γ represents the normalized kernel bandwidth.

#### 2.1.5 LncRNA functional similarity

We adopt the method proposed by Sun et al. (2014) to calculate the functional similarity of lncRNAs(LFS). The formula is as follows:

$$LFS\left(l_{i},l_{j}\right)=\frac{\sum_{1\leq i\leq nD_{i}}SS\left(d_{i},D_{i}\right)+\sum_{1\leq i\leq nD_{j}}SS\left(d_{j},D_{j}\right)}{nD_{i}+nD_{j}}$$

Where d represents a disease associated with a lncRNA l ; D represents a group of diseases associated with l and nD represents the total number of diseases in this group; $SS\left(d,D\right)$ represents the maximum semantic similarity between d and D .

#### 2.1.6 LncRNA sequence similarity

We are inspired by Li et al. (2020) to introduce lncRNA sequence similarity (LSES), which is calculated using the following formula:

$$LSES\left(l_{i},l_{j}\right)=\frac{cost\left(l_{i},l_{j}\right)}{len\left(l_{i}\right)+len\left(l_{j}\right)}$$

Where $len\left(l\right)$ represents the length of the sequence l ; $cost\left(l_{i},l_{j}\right)$ is used to measure the minimum cost required to transform the sequence of $l_{i}$ into the sequence of $l_{j}$ by performing three types of operations: insertion, deletion, and replacement (with insertion or deletion cost being 1, and replacement cost being 2).

### 2.2 Methods

**FIGURE 2:**
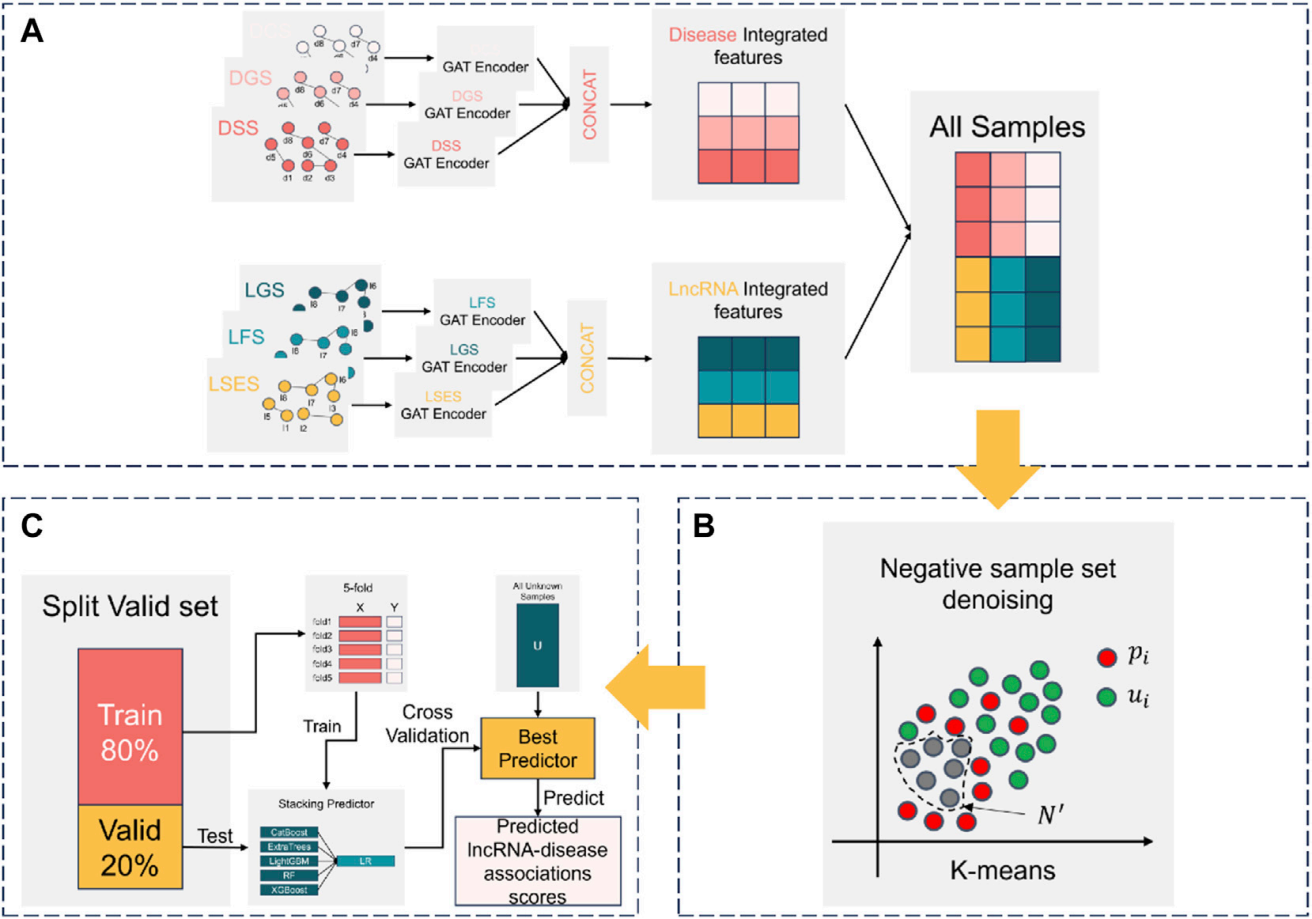
The construction process of NDMLDA comprises three parts: **(A)** multi-view feature extraction; **(B)** negative sample set denoising; **(C)** training and prediction of the stacking ensemble classifier.

The construction process of NDMLDA is shown in Figure 2, which mainly consists of three steps (A) multi-view feature extraction; (B) negative sample set denoising; (C) training and prediction of the stacking ensemble classifier.

#### 2.2.1 Multi-view feature extraction

**FIGURE 3:**
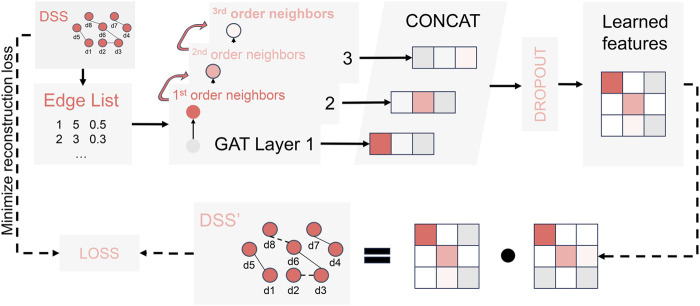
The encoding process of a specific input network.

The Graph Attention Network (GAT) has demonstrated significant potential in predicting LDA as a primary approach for multi-view feature extraction (Shi et al., 2021; Liang et al., 2022; Zhao et al., 2022) Following the guidance of previous literature (Forster et al., 2022), we developed a GAT-based module for multi-view feature extraction, as depicted in Figure 3. To begin with, we transformed the similarity networks of various views (including DSS, DCS, DGS, LFS, LGS, and LSES) into edge list format, where each row represents the source, target, and weight. Subsequently, for each input network, we constructed an encoder by concatenating three GAT layers, as illustrated in Figure 3. This encoding process enables the learning of high-order neighborhood features for the specific input networks using dedicated encoders. Next, by employing feature aggregation and random loss processing, a unified disease (or lncRNA) feature H is generated. Finally, all node features were arranged in a matrix F . Through multiple experiments, we fixed the length of this feature to 64 (see supplementary material for details).

$$GAT\left(A,H\right)=\sigma \left(\alpha HW^{T}\right)$$

Where α represents the attention coefficient, H represents the features of nodes in A , W represents the trainable weight parameters, T represents the transpose operation, and σ represents the non-linear activation function LeakyReLU (Maas et al., 2013).

To enhance the quality of feature extraction, we decode and reconstruct the unified feature matrix F , which has learned the lncRNA (or disease). Our objective is to minimize the discrepancy between the reconstructed network $F^{T}$ and the original input network. The process of network reconstruction after decoding is exemplified below.

$$A=F∙F^{T}$$

The loss function in this process can be defined as follows:

$$Loss=\frac{1}{n^{2}}\sum_{j}^{N}{\left|\left|b_{j}⊙\left(A-A_{j}\right)⊙b_{j}^{T}\right|\right|}_{F}^{2}$$

Where, n represents the total number of nodes in the input network, $b_{j}$ represents the node mask in input network j , $A_{j}$ represents the adjacency matrix corresponding to input network j , ⊙ represents the inner product and ${\left\|∙\right\|}_{F}$ represents the F-norm.

#### 2.2.2 Negative sample set denoising

**FIGURE 4:**
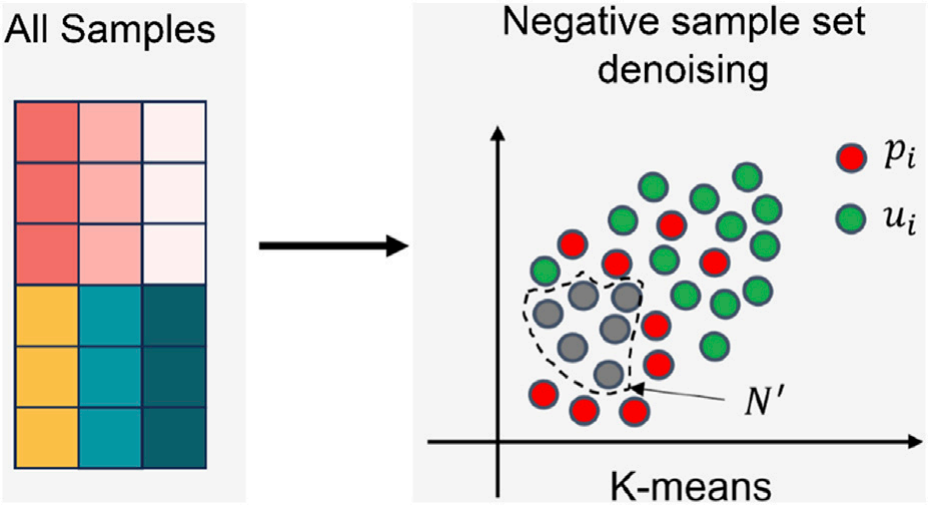
The denoising process of negative sample set.

The process of the negative sample set denoising is shown in Figure 4 Firstly, the positions of elements with values 1 and 0 in the LDA matrix are recorded separately. Then, the unified feature vectors of corresponding diseases and lncRNAs are retrieved based on these positions. These two feature vectors are directly concatenated, with diseases preceding lncRNAs, to form a sample. The complete sample set (All Samples) is obtained by concatenating the features of all positions. Next, the number of clusters K is determined by calculating the silhouette coefficient. The silhouette coefficient (SC), which ranges from −1 to 1, is a commonly used indicator in previous studies for evaluating the effectiveness of clustering algorithms (Rousseeuw, 1987).

SC usually follows the trend of K-value changes. When the silhouette coefficient approaches 1, the K-value also approaches the ideal value. SC can be calculated as follows:

$$SC_{i}=\frac{C_{b}\left(i\right)-C_{a}\left(i\right)}{maximum\left(C_{a}\left(i\right),C_{b}\left(i\right)\right)}$$

Where, $C_{a}\left(i\right)$ represents the average distance between sample i and the other samples in its cluster, while $C_{b}\left(i\right)$ represents the minimum average distance between sample i and the samples in different clusters. In this study, we set K as 3.

We used the K-means algorithm (Hartigan and Wong, 1979) to perform 10 rounds of clustering on the entire sample set. The complete description of the negative sample denoising process is as follows:

Let P represent the known positive sample set, $P=\left\{p_{1},p_{2},\ldots ,p_{m}\right\}$ , where each sample $p_{i}$ represents a known lncRNA-disease association. Let U represent the unknown sample set, $U=\left\{u_{1},u_{2},\ldots ,u_{n-m}\right\}$ . Assuming that the samples in U that are similar to P are noise samples, we take the following steps to denoise U :

First, we cluster the entire sample set using the K-means algorithm, which results in cluster divisions $C=\left\{C_{1},C_{2},\ldots ,C_{k}\right\}$ , where each cluster $C_{i}$ is a set. For each cluster $C_{i}$ , we calculate the proportion of positive samples and denote it as $r\left(C_{i}\right)$ .

Then, we repeat the following steps 10 times:

1. Cluster the sample set using the K-means algorithm to obtain cluster divisions $C^{\prime }=\left\{C_{1}^{\prime },C_{2}^{\prime },\ldots ,C_{k}^{\prime }\right\}$ .
2. For each cluster $C_{i}^{\prime }$ , calculate the proportion of positive samples and denote it as $r^{\prime }\left(C_{i}^{\prime }\right)$ .
3. Find the cluster $C_{i}^{\prime }$ with the highest $r^{\prime }\left(C_{i}^{\prime }\right)$ and denote its unknown sample set as $U^{\prime }$ .
4. Save $U^{\prime }$ .

Finally, we take the intersection of the noise sample sets obtained from these 10 clustering iterations, $U_{noise}=U_{1}^{\prime }\cap U_{2}^{\prime }\cap \ldots \cap U_{10}^{\prime }$ , and remove these samples from U . The final denoised unknown sample set is represented as $U_{reliable}=U-U_{noise}$ . The unknown samples in $U_{reliable}$ represent the denoised negative samples.

#### 2.2.3 Training stacking ensemble classifier

**FIGURE 5:**
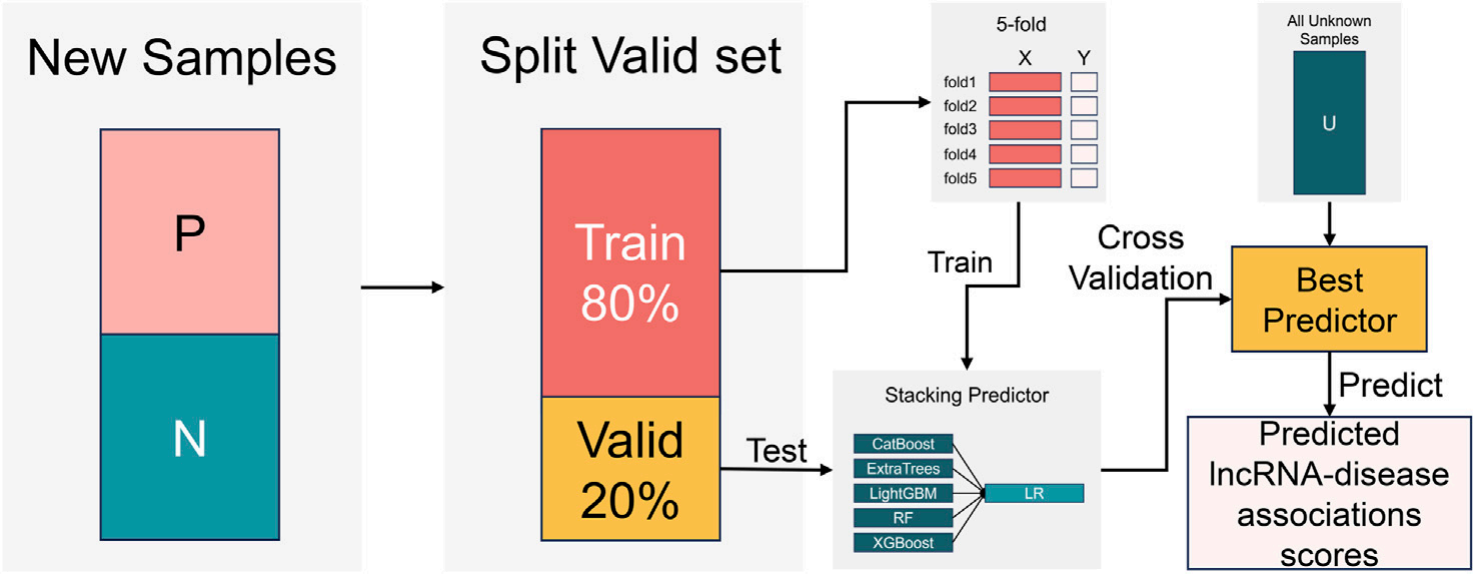
The training process of a stacking ensemble classifier.

To overcome the limited predictive capabilities of individual classifier, we draw inspiration from previous research (Li et al., 2021; Liang et al., 2022). The training process of the stacking ensemble classifier is illustrated in Figure 5. Five decision tree-based classifiers, including CatBoost (Dorogush et al., 2018), ExtraTrees (Geurts et al., 2006), LightGBM (Ke et al., 2017), RandomForest (Breiman, 2001), and XGBoost (Chen and Guestrin, 2016), are employed as base classifier, with LogisticsRegression (Cramer, 2002) serving as the meta-classifier. This framework creates a stacked ensemble LDA prediction model (refer to the supplementary material for the training process of the ensemble classifier). We conduct a five-fold cross-validation on 80% of the samples from the reconstructed new dataset (details can be found in the supplementary material), while the remaining 20% of samples are used as an independent dataset to evaluate the trained classifiers. Finally, we select the classifier with the best performance for the final LDA prediction.

## 3 Results

### 3.1 Experimental settings

The performance evaluation of NDMLDA is conducted using five performance metrics: accuracy (ACC), Matthew’s correlation coefficient (MCC) (Harald, 1946), F1-score, area under the receiver operating characteristic curve (AUC), and area under the precision-recall curve (AUPR). The calculation formulas for these metrics are as follows:

$$ACC=\frac{TN+TP}{TN+TP+FN+FP}$$

$$MCC=\frac{TP\times TN-FP\times FN}{\sqrt{\left(TP+FN\right)\times \left(TP+FP\right)\times \left(TN+FN\right)\times \left(TN+FP\right)}}$$

$$F1-score=\frac{2\times Precision\times Recall}{Precision+Recall}$$

$$Precision=\frac{TP}{TP+FP}$$

$$Recall=\frac{TP}{TP+FN}$$

In the context of the confusion matrix, TP, TN, FP, and FN are variables that represent the four different types of prediction situations.

### 3.2 Comparison results with other methods

We conducted a comparative analysis of several LDA prediction methods, including MAGCNSE (Liang et al., 2022), MCHNLDA (Zhao et al., 2022), VGAELDA (Shi et al., 2021), CapsNet-LDA (Zhang et al., 2022), LDAformer (Zhou et al., 2022), and SSMF-BLNP (Xie et al., 2023).

MAGCNSE (Liang et al., 2022) employs a two-step approach, first utilizing GCN to extract the multi-view representation of lncRNA and diseases, and then employing CNN to obtain the final representation. The integrated classifier is then used for prediction (Zhao et al., 2022).

VGAELDA (Shi et al., 2021) proposes a LDA prediction method that combines variational inference and graph autoencoders.

CapsNet-LDA (Zhang et al., 2022) presents a prediction method that leverages capsule networks and stacked autoencoders.

LDAformer (Zhou et al., 2022) introduces a LDA prediction method based on topological feature extraction and Transformer encoding.

As shown in Table 1, NDMLDA achieved higher AUC and AUPR by 2.5% and 1.6%, respectively, compared to the second-best MAGCNSE. Furthermore, the overall performance of NDMLDA (with all metrics above 92%) is superior to other comparative methods.

**TABLE 1:** Comparison of the performance of NDMLDA with other LDA prediction methods.

Model	AUC	AUPR	MCC	F1	ACC
NDMLDA	0.9907 ± 5.2e-8	0.9927 ± 2.2e-8	0.9249 ± 9.6e-6	0.9631 ± 2.3e-6	0.9624 ± 2.4e-6
NDMLDA^*1^	0.9683 ± 2.9e-7	0.9718 ± 2.7e-7	0.8229 ± 2.2e-5	0.9135 ± 4.9e-6	0.9114 ± 5.3e-6
MAGCNSE	0.9665 ± 4.8e-6	0.9773 ± 1.26e-5	0.8729 ± 9.4e-5	0.9462 ± 1.4e-4	0.9357 ± 1.2e-7
VGAELDA	0.9212 ± 3.1e-4	0.7469 ± 1.2e-4	0.6872 ± 5.3e-4	0.6514 ± 8.9e-4	0.9806 ± 1.4e-6
CapsNet-LDA	0.9634 ± 1.5e-5	0.7452 ± 1.2e-5	0.6764 ± 9.9e-5	0.6843 ± 8.2e-5	0.9836 ± 9.5e-7
LDAformer	0.9452 ± 9.3e-4	0.2439 ± 1.2e-2	0.1844 ± 5.5e-3	0.1034 ± 1.2e-3	0.9403 ± 7.4e-4
SSMF-BLNP	0.8251 ± 1.1e-5	0.1535 ± 3e-5	0.1815 ± 2e-5	0.4339 ± 2e-5	0.9255 ± 1.4e-5

^1^NDMLDA^*^

Stands for negative sample denoising not being executed.

MAGCNSE and CapsNet-LDA mitigate the impact of sparse features on the model through a multi-view approach, achieving good performance (overall performance higher than 0.8). However, they are affected by negative sample noise, resulting in suboptimal performance. Additionally, as shown in Table 1, our model, despite having a decrease in performance in five evaluation metrics without sample reconstruction, still outperforms methods such as SSMF-BLNP and CapsNet-LDA. This indicates that our negative sample denoising module is effective in mitigating the impact of negative sample noise on the model.

LDAformer proposed a method for LDA prediction based on topological feature extraction and Transformer encoder. By enhancing feature extraction, the performance of complex models is improved. Compared to our method, without using the sample denoising module, we obtain multi-view features through GAT and achieve better overall performance in LDA using a simple stacking model. This indicates that our multi-view feature processing method is effective.

Meanwhile, to further demonstrate the generalization ability of our method, we conducted comparative experiments on an independent dataset lncTarD. The experimental results are shown in Table 2. It can be observed that our proposed method still outperforms the comparative methods in four main indicators, indicating the robustness of NDMLDA.

**TABLE 2:** Comparison of the performance of NDMLDA with other LDA prediction methods on lncTarD dataset.

Model	AUC	AUPR	MCC	F1	ACC
NDMLDA	0.9479 ± 1.5e-4	0.9635 ± 1.6e-4	0.7852±1e-3	0.8917 ± 4.7e-4	0.8928 ± 2.4e-4
MAGCNSE	0.9492 ± 1.9e-5	0.7314 ± 9.36e-4	0.6439 ± 8.6e-4	0.6279 ± 1e-3	0.9857 ± 9.7e-7
VGAELDA	0.9337 ± 3.9e-4	0.7779 ± 1.5e-3	0.7825 ± 9.6e-4	0.7637 ± 1.3e-3	0.9903 ± 1.5e-6
CapsNet-LDA	0.9058 ± 3e-4	0.7367 ± 1.2e-3	0.7312 ± 1e-3	0.7262 ± 1e-3	0.9896 ± 9.5e-7
LDAformer	0.8115 ± 1.1e-3	0.2574 ± 5e-4	0.1937 ± 7.5e-5	0.0415 ± 2.9e-5	0.8527 ± 3e-3
SSMF-BLNP	0.9303 ± 1.1e-5	0.5738 ± 1e-4	0.3791 ± 3.1e-5	0.7936 ± 5.3e-5	0.943 ± 2.4e-4

### 3.3 Ablation studies

#### 3.3.1 The influence of negative sample set denoising on the predictive performance of NDMLDA

**FIGURE 6:**
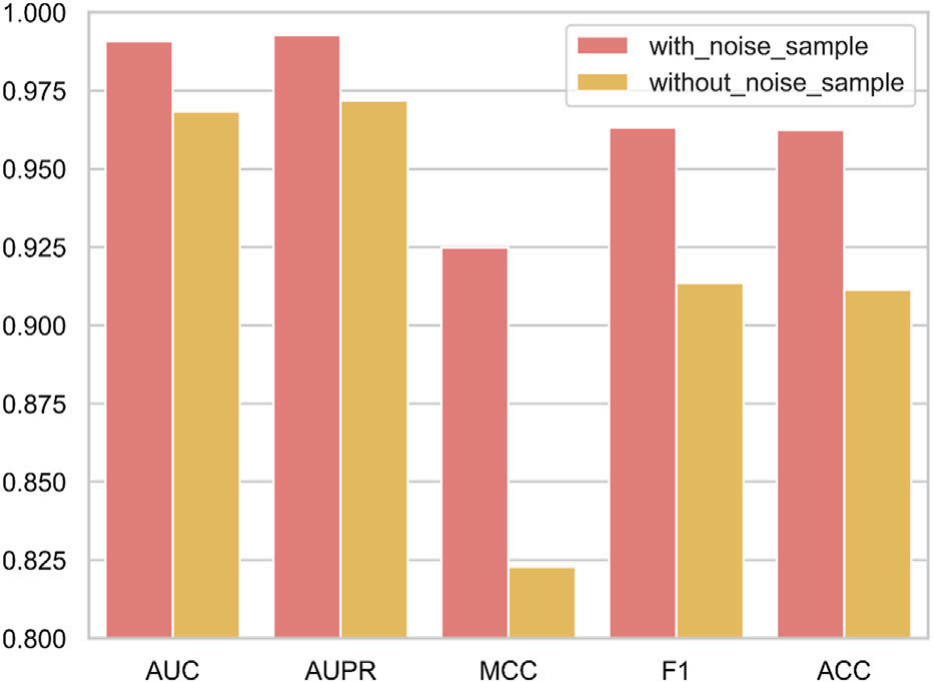
The influence of negative sample set denoising module on the predictive performance of NDMLDA.

**FIGURE 7:**
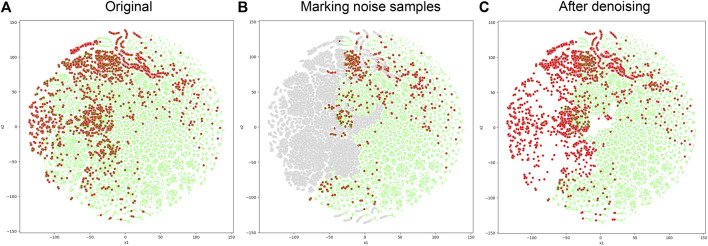
Comparison of sample distribution before and after negative sample denoising. **(A)** represents the original distribution of samples, with red dots indicating positive samples and green dots indicating unknown samples; **(B)** uses gray dots to represent noisy samples in the unknown samples; **(C)** represents the distribution of samples after denoising.

When the negative sample set denoising module is integrated into NDMLDA (as depicted in Figure 6), all five performance measures exhibit superior results compared to the state without the module. Notably, the addition of the module improves the AUC by 2.3%, AUPR by 2.2%, MCC by 12.4%, F1-score by 5.4% and ACC by 5.6%. These findings suggest that incorporating the negative sample set denoising module enhances the prediction performance of NDMLDA. We visualized the distribution of samples before and after denoising using t-SNE (Maaten and Hinton, 2008). Figure 7 shows the visualization results. Comparing Figures 7A,C, it can be observed that our proposed method for denoising the negative sample set successfully removes the noisy samples.

#### 3.3.2 Classifier selection

Table 3 demonstrates that among the five metrics, the stacked ensemble classifier attained optimal results for three of them. While the stacked ensemble classifier’s performance in terms of MCC and ACC is slightly lower than that of ExtraTrees (with a maximum difference of 0.02%), it surpasses ExtraTrees in the more significant evaluation metrics of AUC and AUPR (with improvements of 0.16% and 0.15% respectively). These results indicate that the inclusion of the stacked ensemble classifier can enhance the predictive performance of NDMLDA.

**TABLE 3:** The performance comparison between individual classifiers and stacked ensemble classifiers.

Classifier	AUC	AUPR	MCC	F1	ACC
Stacking	0.9907 ± 5.2e-8	0.9927 ± 2.2e-8	0.9249 ± 9.6e-6	0.9631 ± 2.3e-6	0.9624 ± 2.4e-6
LogisticsRegression	0.9825 ± 9.6e-9	0.9845 ± 1.2e-8	0.8721 ± 1.3e-	0.9372 ± 3.9e-7	0.9360 ± 3.5e-7
RandomForest	0.9890 ± 3.3e-7	0.9914 ± 1.1e-7	0.9178 ± 5.9e-6	0.9597 ± 1.4e-6	0.9590 ± 1.5e-6
ExtraTrees	0.9891 ± 3.3e-7	0.9912 ± 1.9e-7	0.9251 ± 6.9e-6	0.9579 ± 1.4e-6	0.9625 ± 1.7e-6
XGB	0.9904 ± 2.4e-7	0.9924 ± 1.2e-7	0.9143 ± 9.7e-6	0.9582 ± 2.2e-6	0.9571 ± 2.4e-6
LGBM	0.9907 ± 1.4e-7	0.9924 ± 5.4e-8	0.9096 ± 1.2e-5	0.9559 ± 2.9e-6	0.9548 ± 3.1e-6
MLP	0.9887 ± 1.5e-6	0.9907 ± 1.5e-6	0.9006 ± 9.9e-5	0.9536 ± 1.8e-5	0.9503 ± 2.4e-5
SVM	0.9855 ± 5.4e-8	0.9882 ± 1.8e-8	0.8914 ± 6.2e-7	0.9465 ± 1.7e-7	0.9456 ± 1.7e-7

#### 3.3.3 Combination of different views

**FIGURE 8:**
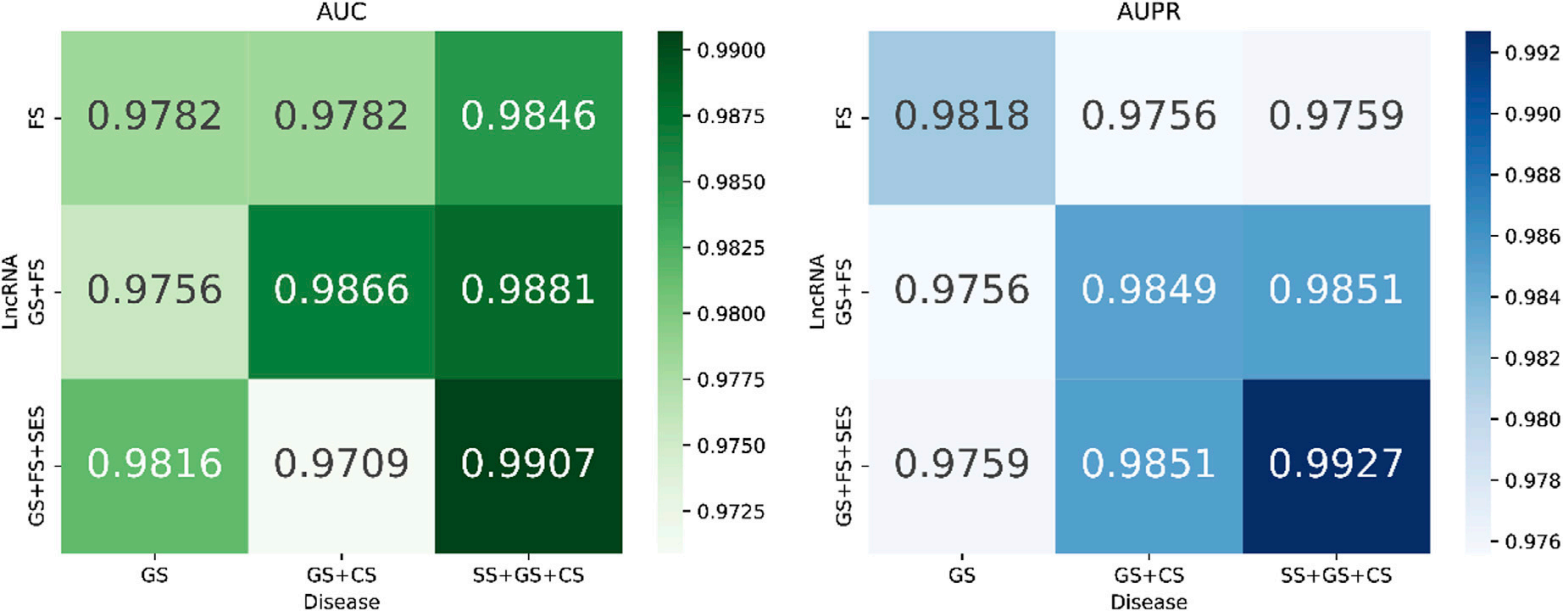
AUC and AUPR corresponding to different combinations of views.

According to Figure 8, the performance of the model is influenced by the combination of different views (AUC: 0.9709–0.9907; AUPR: 0.9818–0.9927). Furthermore, increasing the number of combined views leads to an improvement in the model’s performance. To construct a more precise LDA prediction model, we have chosen to utilize fusion features from lncRNA, which include lncRNA gaussian interaction profile kernel similarity (LGS), lncRNA functional similarity (LFS), and lncRNA sequence similarity (LSES), as well as fusion features from diseases, which include disease semantic similarity (DSS), disease gaussian interaction profile kernel similarity (DGS), and disease cosine similarity (DCS).

### 3.4 Case studies

To further validate the performance of NDMLDA in predicting the association between specific diseases and lncRNA, we conducted case studies on six prevalent cancers: breast cancer, cervical cancer, colon cancer, esophageal cancer, lung cancer, and stomach cancer. In each case study, we utilized all samples related to cancer as the testing set, while the remaining samples served as the training set. Subsequently, we trained NDMLDA on the training set and employed it to evaluate the samples in the testing set.

The validated lncRNAs related to breast cancer and cervical cancer are summarized in Table 4 and Table 5, respectively. In the evidence column, “C” denotes candidate lncRNAs corroborated by the Lnc2Cancer database. “D” denotes candidate lncRNAs supported by the LncRNADisease database. “P” denotes candidate lncRNAs supported by a single literature source. “R” denotes candidate lncRNAs corroborated by the RNADisease database. “P*” denotes candidate lncRNAs supported by multiple published literature sources. Further details regarding the predictions of NDMLDA for lncRNAs associated with four other cancers can be found in the supplementary materials.

**TABLE 4:** Top 30 lncRNAs related to breast cancer predicted by NDMLDA.

No.	LncRNA	Evidence	No.	LncRNA	Evidence
1	TUNAR	P&R&D	16	SNHG3	P^*^&R&C&D
2	SLC26A4-AS1	P&R&D	17	DLX6-AS1	P^*^&R&C&D
3	LINC00665	P^*^&R&C&D	18	LINC00319	P&R
4	HAR1B	P&R&D	19	NEAT1	P^*^&R&C&D
5	SNHG15	P^*^&R&C&D	20	MCM3AP-AS1	P&R&D
6	KCNQ1OT1	P^*^&R&C&D	21	DLEU1	P&R&C&D
7	DPP10-AS1	P&R&D	22	HMMR-AS1	P&R&C&D
8	LINC00461	P^*^&R&C&D	23	SNHG7	P^*^&R&C&D
9	FEZF1-AS1	P^*^&R&C	24	LINC00339	P&R&D
10	HOXA11-AS	P^*^&R&C&D	25	MIR7-3HG	P&R&D
11	SNHG4	P&R&D	26	ZFAS1	P^*^&R&C&D
12	FAS-AS1	P^*^&R&C	27	OIP5-AS1	P^*^&R&C&D
13	FENDRR	P&R&C&D	28	GNG12-AS1	P&R&C&D
14	ST8SIA6-AS1	P^*^&R&C&D	29	LINC01234	P^*^&R&D
15	FOXC2-AS1	P&R&C&D	30	PAX8-AS1	P&R&D

We systematically validated the top 30 lncRNAs, associated with each specific type of cancer by cross-referencing three important databases: LncRNADisease v2.0, Lnc2Cancer v3.0, and RNADisease v4.0 (Bao et al., 2019; Gao et al., 2021; Chen et al., 2022), as well as consulting relevant literature records.

**TABLE 5:** Top 30 lncRNAs related to cervical cancer predicted by NDMLDA.

No.	LncRNA	Evidence	No.	LncRNA	Evidence
1	SNHG8	P&R&C&D	16	PCGEM1	P&R&C&D
2	LINC00665	P&R	17	LINC01503	P^*^&R&C&D
3	TDRG1	P^*^&R&C&D	18	ZFAS1	P^*^&R&C&D
4	GAS5-AS1	P&R&C&D	19	OIP5-AS1	P^*^&R&C&D
5	FEZF1-AS1	P^*^&R&C	20	PAX8-AS1	P^*^&R&D
6	HOXA11-AS	P^*^&R&C&D	21	BDNF-AS	P&R
7	SNHG4	P&R&C&D	22	LINC01139	P&R&C&D
8	FENDRR	P&R&D	23	MIR22HG	P^*^&R&C&D
9	SNHG3	P&R&C&D	24	TUSC8	P^*^&R&C& D
10	DLG1-AS1	P&R&C&D	25	FOXD2-AS1	P^*^&R&C&D
11	DLX6-AS1	P^*^ & R & C &D	26	CYTOR	P^*^ & R & D
12	LINC00319	P&R&C&D	27	MALAT1	P^*^&R&C&D
13	NEAT1	P^*^&R&C&D	28	PCAT6	P^*^&R&C&D
14	DLEU1	P&R&C&D	29	SOX2-OT	P^*^&R&D
15	SNHG7	P^*^&R&C&D	30	PVT1	P^*^&R&C&D

## 4 Discussion

The NDMLDA method utilizes the negative sample denoising module to obtain negative sample data that closely approximates the real distribution. Instead of introducing a new clustering method, our approach focuses on integrating the multi-view similarity network with the negative sample denoising technique. To achieve this, we adopt the K-means algorithm, a well-established clustering algorithm, as the core algorithm for negative sample denoising.

The NDMLDA model demonstrates good performance by combining stacked classifiers. However, we have also noticed that several single classifiers used for comparison have AUC and AUPR values around 0.99. On one hand, this is because we balanced the positive and negative samples during classifier evaluation. On the other hand, it is due to the relatively small number of known lncRNA-disease associations, which results in an insufficient number of samples for performance evaluation. However, considering the increasing complexity of data in future model applications, we have chosen the stacked ensemble classifier as our final classifier to ensure the competitiveness of our model.

However, our proposed model (NDMLDA) still has some limitations. Although we obtained a large number of known LDAs (8,334) by merging multiple databases, the comparison with the huge number of unknown samples (313,085) is still very sparse. At the same time, the dataset only includes a limited number of lncRNA-disease pairs, which is only a small fraction of the real-world scenarios. Therefore, in the future, we will attempt to further expand the number of LDAs in the dataset to address the constantly changing real situations. We also recognize that there is still a possibility that some reliable negative samples may be discarded in the process. To mitigate this, we plan to conduct further research and improvements in our future work.

LncRNAs have been established as pivotal regulators of gene expression, playing a significant role in a wide range of biological functions and disease processes, including cancer. This study presents a model known as NDMLDA, which integrates multi-view feature extraction, unsupervised negative sample denoising, and stacked ensemble classifier. The experimental results demonstrate that the proposed prediction method achieves exceptional performance across five metrics (including AUC, AUPR, MCC, F1-score and ACC). Additionally, the accuracy and reliability of NDMLDA in the prediction process for LDA are further substantiated through six case studies (involving breast cancer, cervical cancer, colon cancer, esophageal cancer, lung cancer, and gastric cancer).

## 5 Conclusion

This article introduces an LDA prediction model (NDMLDA) that combines negative sample denoising and multi-view network feature extraction. The experimental results demonstrate that our method outperforms the six recent base models, achieving excellent performance in five metrics (including AUC, AUPR, and MCC). Additionally, the results of six case studies (breast cancer, cervical cancer, colon cancer, esophageal cancer, lung cancer, and gastric cancer) further validate the accuracy and reliability of NDMLDA in LDA prediction tasks.

## Data Availability

The original contributions presented in the study are included in the article/Supplementary Material, further inquiries can be directed to the corresponding author.
